# Cryo‐EM Structures of Native Chromatin Units From Human Cells

**DOI:** 10.1111/gtc.70019

**Published:** 2025-04-14

**Authors:** Suguru Hatazawa, Yoshiyuki Fukuda, Yuki Kobayashi, Lumi Negishi, Masahide Kikkawa, Yoshimasa Takizawa, Hitoshi Kurumizaka

**Affiliations:** ^1^ Laboratory of Chromatin Structure and Function Institute for Quantitative Biosciences, The University of Tokyo Tokyo Japan; ^2^ Division of Molecular CytoMorphology Institute of Advanced Medical Sciences, Tokushima University Tokushima Tokushima Japan; ^3^ Department of Cell Biology and Anatomy, Graduate School of Medicine The University of Tokyo Tokyo Japan; ^4^ Department of Computational Biology and Medical Sciences, Graduate School of Frontier Sciences The University of Tokyo Tokyo Japan; ^5^ Department of Biological Sciences, Graduate School of Science The University of Tokyo Tokyo Japan; ^6^ RIKEN Center for Biosystems Dynamics Research Yokohama Japan

**Keywords:** chromatin, cryo‐electron tomography, cryo‐EM, nucleosome, single particle analysis

## Abstract

In eukaryotic cells, genomic DNA is compacted by nucleosomes, as basic repeating units, into chromatin. The nucleosome arrangement in chromatin fibers could be an important determinant for chromatin folding, by which genomic DNA is regulated in the nucleus. To study the structures of chromatin units in cells, we have established a method for the structural analysis of native mono‐ and poly‐nucleosomes prepared from HeLa cells. In this method, the chromatin in isolated nuclei was crosslinked to preserve the proximity information between nucleosomes, followed by chromatin fragmentation by micrococcal nuclease treatment. The mono‐ and poly‐nucleosomes were then fractionated by sucrose gradient ultracentrifugation, and their structures were analyzed by cryo‐electron microscopy. Cryo‐electron microscopy single particle analysis and cryo‐electron tomography visualized a native nucleosome structure and secondary nucleosome arrangements in cellular chromatin. This method provides a complementary strategy to fill the gap between in vitro and in situ analyses of chromatin structure.

## Graphical abstract

We have established a method for the cryo‐EM structural analysis of native mono‐ and poly‐nucleosomes extracted from human cells. Single particle cryo‐EM and cryo‐electron tomography analyses have visualized a native nucleosome structure and secondary nucleosome arrangements in cellular chromatin.

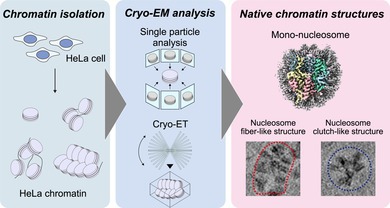

## 1 Introduction

In eukaryotes, the genome is compacted as chromatin, in which nucleosomes are the basic architectural units (Olins and Olins 1974; Kornberg 1974). In the nucleosome, the four histones H2A, H2B, H3, and H4 form heterodimers of H2A–H2B and H3–H4, and two H2A–H2B and H3–H4 dimers associate to form the histone octamer (Richmond et al. 1984; Arents et al. 1991; Luger et al. 1997). This octamer is left‐handedly wrapped with 145–147 base pairs of DNA in the nucleosome (Richmond et al. 1984; Luger et al. 1997).

Chromatin was historically thought to be folded into regular, hierarchical higher‐order structures. The 30 nm fiber was proposed as a model for higher‐order chromatin architecture, as supported by in vitro reconstitution studies under high‐salt conditions (Finch and Klug 1976; Woodcock et al. 1984). Recent technological advances have challenged this classical view. Pioneering cryo‐electron microscopy (cryo‐EM) studies by Eltsov et al. (2008) reported an absence of regular 30 nm fibers in mitotic chromatin (Eltsov et al. 2008). Consistently, the 30 nm fibers were not dominantly observed in human cells when the DNA paths of chromosomes were traced by electron microscopy tomography with a DNA staining fluorescent dye (ChromEMT) (Ou et al. 2017). In situ structural analyses of nucleosomes and poly‐nucleosomes by cryo‐electron tomography (cryo‐ET) have also revealed that chromatin fibers adopt various configurations in the nucleus, and the native and reconstituted nucleosome structures are basically similar (Cai, Böck, et al. 2018). A structural study of isolated mitotic chromosomes by cryo‐ET has also been reported (Beel et al. 2021). However, in these structural analyses with native chromatin fibers, the higher‐order chromatin structure, reflecting the 30 nm fiber, may not be the dominant structure.

These findings are consistent with the previous X‐ray scattering data for native cellular chromatin, again suggesting that the 30 nm fiber may not be the major higher‐order structure of chromatin in mammalian cells (Nishino et al. 2012). Quantitative super‐resolution nanoscopy coupled with computer simulations has visualized “nucleosome clutches,” in which several nucleosomes are heterogeneously associated in the nucleus (Ricci et al. 2015). These results reinforce the emerging view that chromatin exists in a more dynamic and irregular state under physiological conditions.

Using cryo‐focused ion beam (FIB) milling followed by cryo‐ET, chromatin has been visualized in situ in human interphase nuclei (Hou et al. 2023). This approach enables the visualization of chromatin in its native context and has consistently shown that regular 30 nm fibers are not the dominant form of chromatin organization in cells. FIB milling‐based cryo‐ET is powerful but technically demanding and difficult to reproduce, limiting its widespread application.

To overcome these constraints, we established an alternative method for the structural analysis of native chromatin. We prepared native mono‐ and poly‐nucleosomes from human cells under conditions with crosslinking, which preserve the information on spatial association between proximal nucleosomes. We then determined the structures of native human nucleosomes and chromatin units by cryo‐EM single particle analysis and tomography, respectively. This approach provides structural insights comparable to in situ analyses, while being technically more accessible.

## 2 Results

### 2.1 Preparation of Native Chromatin Fragments From HeLa Cells

**FIGURE 1:**
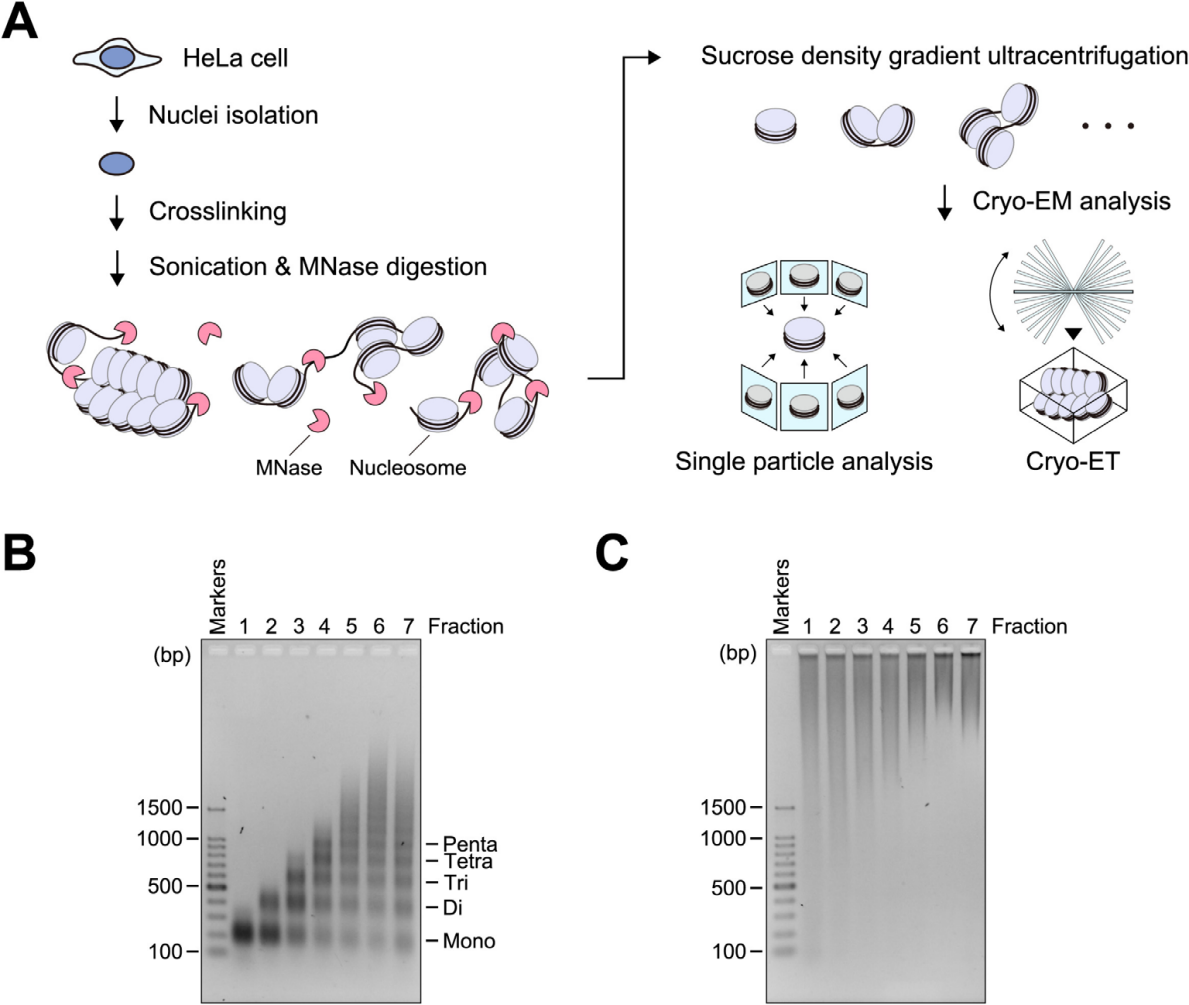
Isolation of chromatin fragments from HeLa cell nuclear extract. (A) Schematic illustration of chromatin fragment isolation. (B and C) Agarose gel electrophoretic analyses of chromatin fragments from HeLa cells. Chromatin samples fractionated by sucrose gradient ultracentrifugation were obtained from top to bottom (lanes 1–7) and analyzed by agarose gel electrophoresis with (B) or without (C) deproteinization. DNA was stained with ethidium bromide.

To prepare chromatin fragments, nuclei were isolated from cultured HeLa S3 cells (Figure 1A). The isolated nuclei were treated with formaldehyde, which forms protein–protein and protein–DNA crosslinks in situ, and the native chromatin was crosslinked to preserve the information of spatial association between proximal nucleosomes. The chromatin was solubilized by sonication and then fragmented by micrococcal nuclease (MNase), which digests the linker regions between nucleosomes. The native chromatin fragments were fractionated by sucrose gradient ultracentrifugation. To assess the DNA lengths in the fractionated chromatin, samples were deproteinized, and the resulting DNA fragments were analyzed using agarose gel electrophoresis. As shown in Figure 1B, the chromatin fragments containing the DNA lengths corresponding to mono‐, di‐, tri‐, tetra‐, penta‐, and larger nucleosome(s) were separated into fractions. To confirm the nucleosome‐nucleosome crosslinking in situ, we analyzed these chromatin fractions without deproteinization (Figure 1C). As anticipated, all chromatin fractions, and even the mono‐nucleosome fraction, contained not only the four core histones but also additional proteins, forming large complexes (Figures 1C and S1A–C). In contrast, the mono‐nucleosome fraction without crosslinking did not form large complexes (Figure S1D). These results suggest that the in situ crosslinking preserves the nucleosome‐nucleosome interactions in nuclei. Therefore, we used chromatin fragments prepared from HeLa cell nuclei with crosslinking for further analysis of the mono‐ and poly‐nucleosome structures.

### 2.2 Cryo‐EM Structure of the Native Nucleosome

**FIGURE 2:**
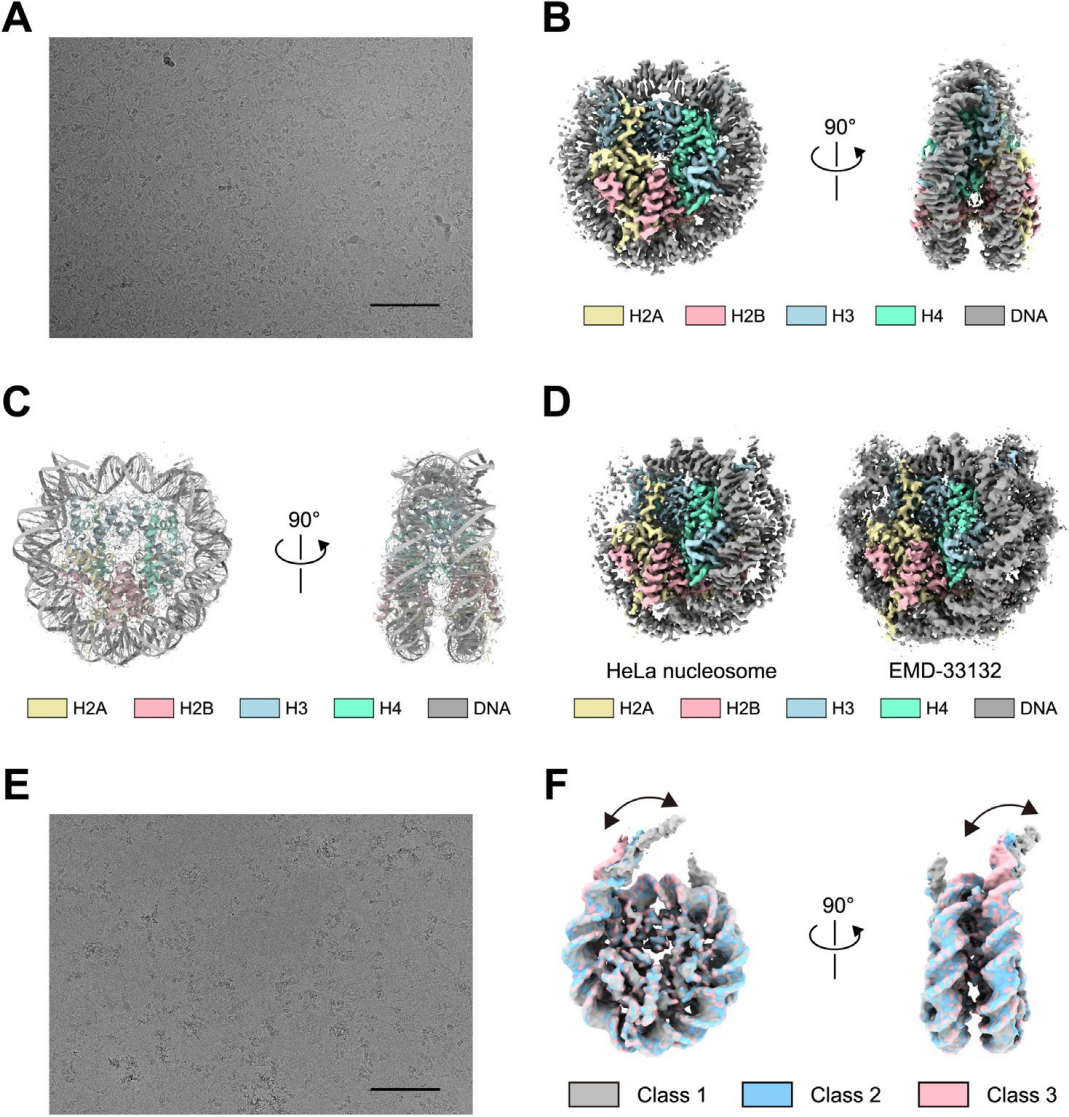
Cryo‐EM structure of the native nucleosome prepared from HeLa cell nuclear extract. (A) Representative cryo‐EM micrograph of native HeLa nucleosomes. The scale bar indicates 100 nm. (B) The cryo‐EM map of the native HeLa nucleosome. Front and side views are presented. (C) An atomic model of the reconstituted nucleosome (PDB ID: 7XD1) was superimposed on the cryo‐EM map of the native HeLa nucleosome. Front and side views are presented. (D) Comparison of the cryo‐EM structure of the native HeLa nucleosome (left) with the cryo‐EM structure of the reconstituted nucleosome with recombinant histones and a Widom 601 DNA fragment (EMD‐33132, right). (E) Representative cryo‐EM micrograph of native HeLa poly‐nucleosomes (Figure 1B, fraction 6). The scale bar indicates 100 nm. (F) Cryo‐EM maps of the native HeLa nucleosome structures obtained from poly‐nucleosomes. Front and side views are presented. The three classes of cryo‐EM maps show different linker DNA orientations.

We first performed the cryo‐EM single particle analysis with the HeLa cell nucleosome. We collected and concentrated the MNase‐digested mono‐nucleosome fraction (Figure S1A) and subjected it to cryo‐EM data collection (Figure S2). In the raw cryo‐EM images, most of the nucleosomes are closely associated, suggesting that the in situ crosslinking preserved the nucleosome‐nucleosome interactions, even in the mono‐nucleosome fraction (Figure 2A).

We then determined the cryo‐EM structure of the HeLa nucleosome by single particle analysis with 1,050,958 particles, at 3.1 Å resolution (Figure 2B and Table S1). In the HeLa nucleosome, the structures of the histones and DNA fit very well with the reconstituted nucleosome structure with recombinant histones and an artificial DNA sequence (PDB ID: 7XD1) (Ai et al. 2022) (Figure 2C). However, the entry‐exit DNA regions of the HeLa nucleosome were less obvious as compared to those of the reconstituted nucleosome (EMD‐33132) (Ai et al. 2022) (Figure 2D), suggesting that the entry‐exit DNA regions are more flexible and/or structurally diverse in cells. Indeed, we observed different classes of nucleosome structures in which the entry‐exit regions of the nucleosomal DNA were apparently distinct (Figure S2B). The structural diversity of the nucleosomal DNA around the entry‐exit regions was also detected in the mono‐nucleosome structures obtained from the single particle analysis with the poly‐nucleosomes (Figures 2E,F and S3). These results are consistent with previous cryo‐EM analyses in human and yeast cells (Cai, Böck, et al. 2018; Cai, Song, et al. 2018).

### 2.3 Structures of Native Poly‐Nucleosomes

**FIGURE 3:**
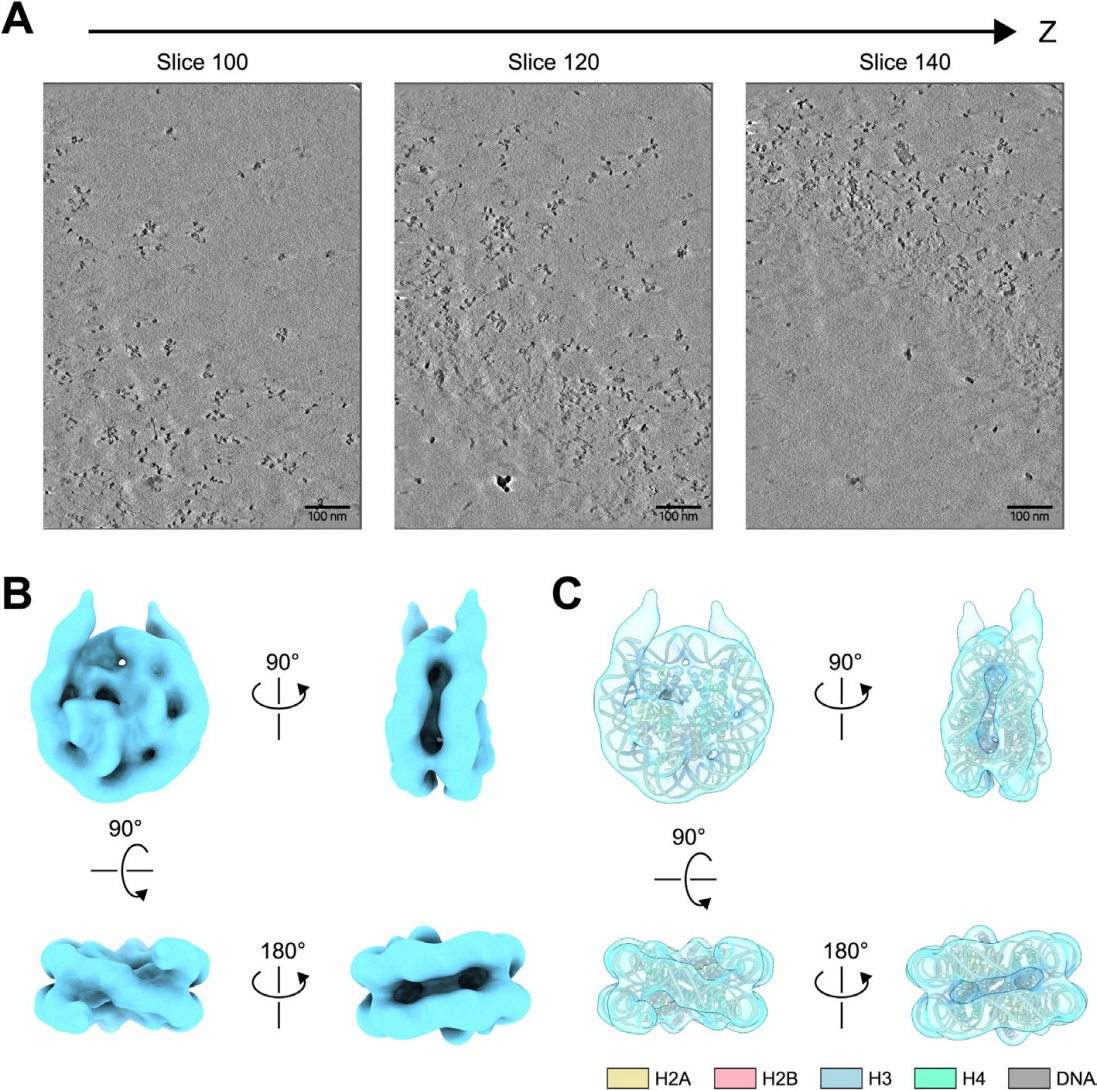
Cryo‐EM structures of native chromatin fragments and subtomogram average of HeLa nucleosome. (A) Representative z‐slice of the tomogram. The scale bar indicates 100 nm. (B) Subtomogram average of the HeLa nucleosome from 1334 particles in five tomograms. (C) Superimposed views of the atomic model of the reconstituted nucleosome (PDB ID: 7VZ4) onto the subtomogram average of the HeLa nucleosome.

We next performed cryo‐ET with poly‐nucleosomes from HeLa cells. To do so, we used penta‐nucleosome and larger HeLa chromatin fragments (Figure 1B, fraction 6). We then obtained cryo‐EM images of native chromatin fragments from HeLa cells and detected chromatin fibers containing various distributions of nucleosome arrangements (Figure 3A). In these chromatin fragments, most of the nucleosomes probably contact the nearest nucleosome by in situ crosslinking, suggesting that these nucleosome arrangements may represent those in the cellular nucleus.

**FIGURE 4:**
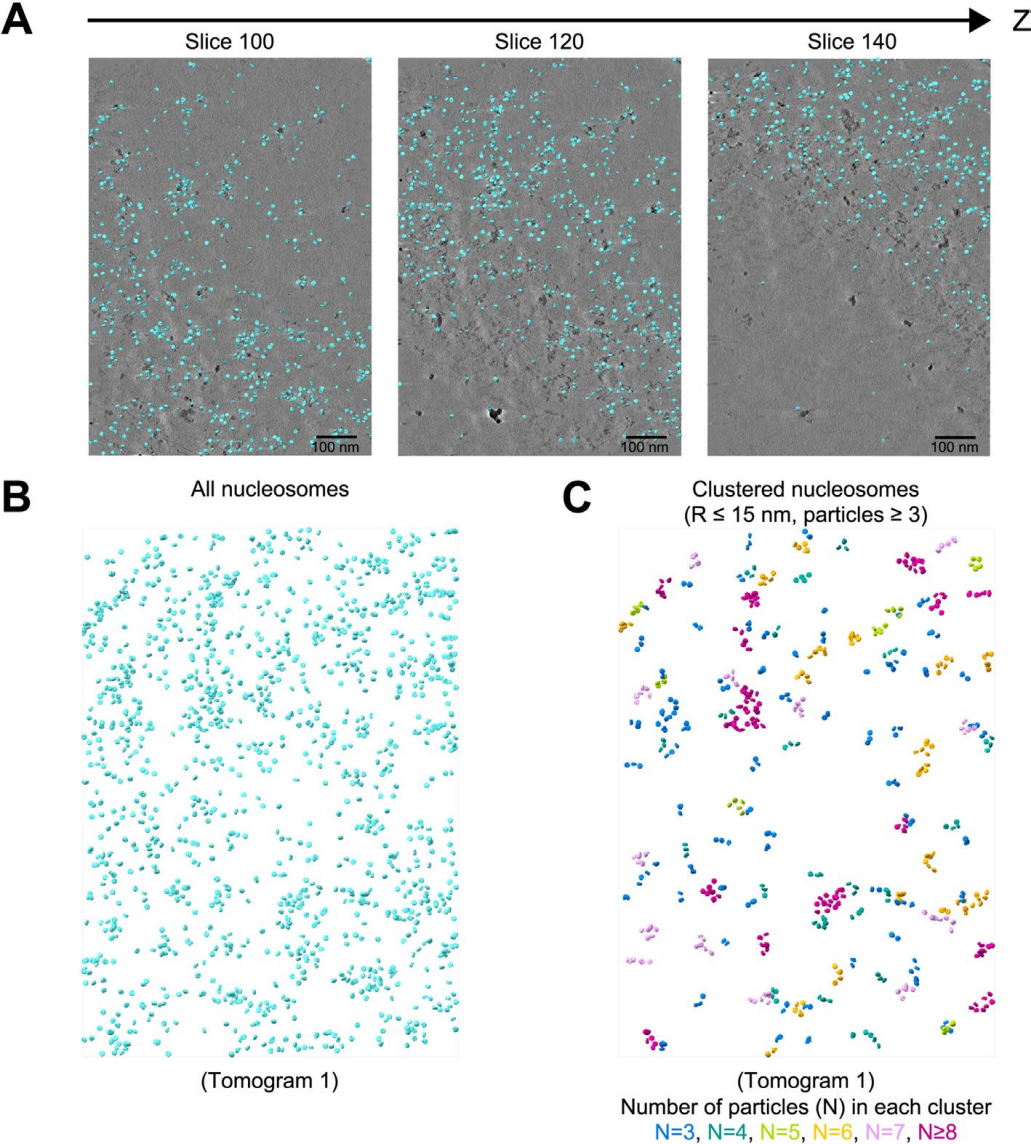
3D organization of nucleosomes in poly‐nucleosomes. (A) Images overlaying z‐slices of the tomogram with mapping back the HeLa nucleosomes in the original tomogram. (B) Mapping back all HeLa nucleosomes in the original tomogram. (C) Mapping back the clustered HeLa nucleosomes in the original tomogram. Each class of nucleosomes is colored according to the number of particles in the cluster.

To obtain an EM map of nucleosome from the tomograms, subtomogram averaging of nucleosome particles detected in tomograms was performed (Figures 3B and S4). Similar to the HeLa nucleosome structures observed from the single particle analysis, the subtomogram average of HeLa nucleosomes fitted well with the atomic model of reconstructed nucleosomes (Figure 3C). To further elucidate the spatial association of nucleosomes in poly‐nucleosomes, the subtomogram averaged structure was pasted back into tomograms at the coordinates detected by a template matching method (Figures 4A,B and S4). The nucleosome particles localized within a 15 nm distance were classed, and each class containing three or more particles was displayed to visualize nucleosome clusters (Figures 4C and S5).

**FIGURE 5:**
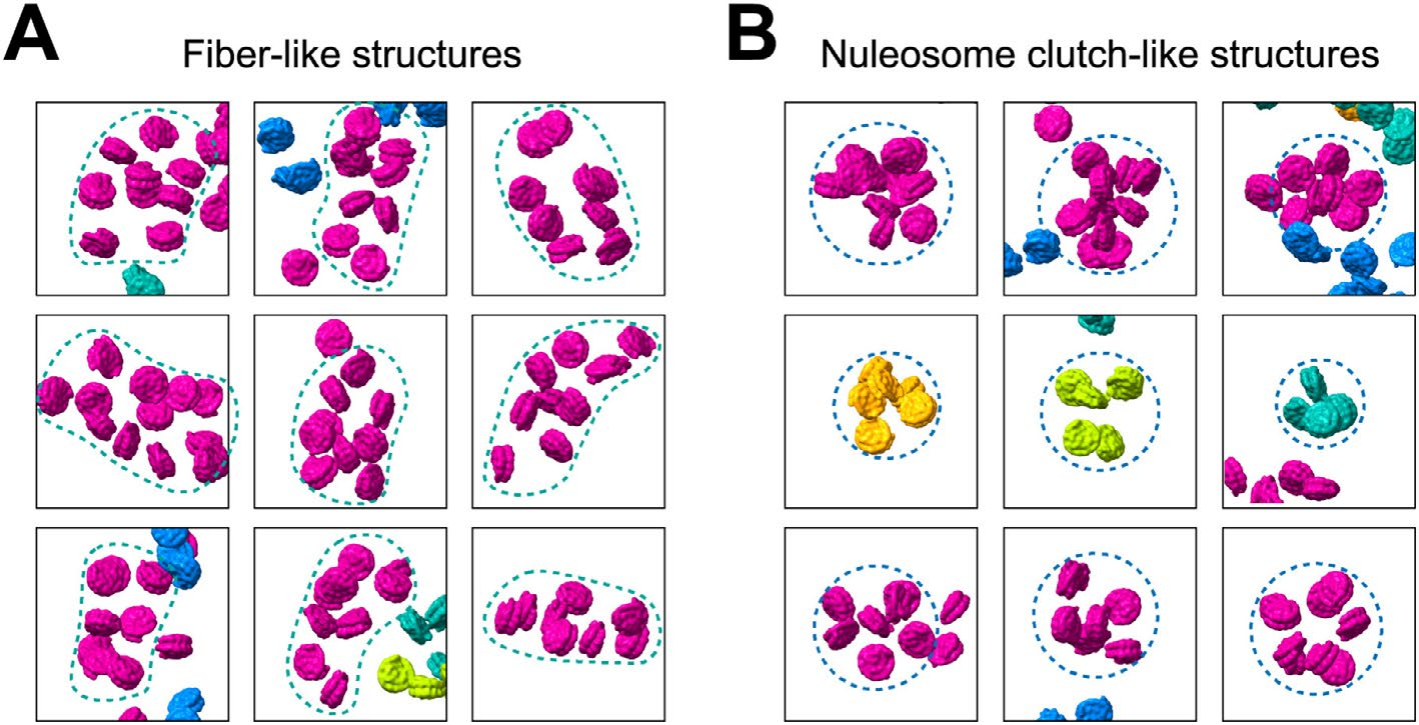
Enlarged views of the mapping back from the tomograms. (A) Fiber‐like and (B) nucleosome clutch‐like structures are surrounded by green and blue dotted lines, respectively.

In previous studies, it was suggested that every other nucleosome is stacked on the disk‐like histone surface in the 30 nm fiber‐like poly‐nucleosomes, and the nucleosomes are connected by linker DNAs with a zig‐zag configuration (Schalch et al. 2005; Song et al. 2014; Takizawa et al. 2020). Therefore, in this study, we defined poly‐nucleosomes as structures containing a stacked nucleosome unit with the zig‐zag configuration. In the cryo‐EM analysis of native chromatin, a structure that could fit with the zig‐zag configuration by nucleosome stacking was detected (Figure 5A). We also visualized heterogeneous nucleosome associations, possibly reflecting the nucleosome clutches (Figure 5B). In contrast, chromatin segments formed by nucleosome association were rarely observed in the cryo‐EM specimen without crosslinking (Figure S6), suggesting that the crosslinking preserves both the fiber‐like and clutch‐like structures of poly‐nucleosomes in the fractionated chromatin samples. The fiber‐like and clutch‐like chromatin architectures were also visualized in a previous in situ cryo‐ET analysis with human interphase nuclei (Hou et al. 2023). Therefore, these structures may provide the proximity information between nucleosomes preserved by crosslinking and could represent the fundamental secondary units of chromatin architecture in eukaryotic cells.

Notably, the poly‐nucleosome fraction analyzed by cryo‐EM contained linker histone H1 (Figure S1C). However, the H1 density could not be clearly observed in the actual tomograms of chromatin, probably due to its flexibility and/or fast exchange (Misteli et al. 2000). Alternatively, the number of particles analyzed may have been insufficient to visualize H1 density. Moreover, the poly‐nucleosomes in this study were solubilized by MNase, which preferentially digests chromatin regions in a relatively open conformation. The absence of detectable H1 density may therefore reflect an open chromatin state, where H1 levels are low.

## 3 Discussion

Nucleosome structures have been determined by X‐ray crystallography and cryo‐EM technology, but most contained artificial nucleosome positioning DNA sequences (Luger et al. 1997; Tachiwana et al. 2010, 2011; Vasudevan et al. 2010; Chua et al. 2012, 2016; Ai et al. 2022). These artificial DNA sequences are advantageous for obtaining homogeneous samples, but they reinforce the DNA‐histone associations, especially at the entry‐exit regions of the nucleosome. In the present study, we determined the cryo‐EM structure of the native HeLa cell nucleosome at 3.1 Å resolution by single particle analysis (Figure 2). This HeLa nucleosome structure must represent an average structure of the various nucleosomes in cells (Figure S2). Despite this heterogeneity, the HeLa nucleosome structure is quite similar to that of the homogeneously reconstituted nucleosome, except for the entry‐exit DNA regions. This fact indicates that the previously reported structure of the nucleosome core is also highly conserved in native nucleosomes in human cells.

In contrast, the entry‐exit regions of the nucleosomal DNA are ambiguous in the native human nucleosome, as compared to those in the reconstituted nucleosome (Figure 2D). The ambiguity of these nucleosomal DNA regions may represent their structural versatility among native nucleosomes (Figures 2F and S2). Consistently, the structural variations around the entry‐exit DNA regions of native nucleosomes from *Xenopus laevis* egg extracts and in human and yeast cells were previously reported (Cai, Böck, et al. 2018; Cai, Song, et al. 2018; Arimura et al. 2021; Hou et al. 2023). The incorporation of histone variants reportedly enhances the flexibility of the nucleosomal DNA ends (Kurumizaka et al. 2021). The entry‐exit regions of the nucleosomal DNA directly dictate the spatial locations of neighboring nucleosomes and may affect the higher‐order association of nucleosomes in chromatin (Takizawa et al. 2018). The nucleosomal DNA end flexibility may also depend on DNA sequences. Further structural studies are required to clarify the contributions of DNA sequences and histone species to the chromatin architecture in cells.

Using a cryo‐ET technique, we determined the structures of chromatin fragments obtained from HeLa cells (Figures 3, 4, 5). In our structures of HeLa chromatin fragments, most of the nucleosomes contacted other nucleosomes, and accordingly, these chromatin architectures may preserve the contact information between nucleosomes in cells. The structures of the HeLa chromatin fragments are not homogeneous, suggesting the structural adaptability of the chromatin fibers in cells. We found fiber‐like and nucleosome clutch‐like architectures in the chromatin fragments (Figure 5A,B), which may be representative secondary structural elements for chromatin folding in the nucleus. It will be intriguing to study the structural basis for the formation of these secondary chromatin structures, which may depend on the DNA sequence, histone variants, histone modifications, the cell cycle, and/or stress responses.

The native poly‐nucleosome structures observed in this in vitro analysis are mostly consistent with those found in the nuclear lamella prepared by FIB milling and analyzed by scanning electron microscopy (Hou et al. 2023). This fact indicates that our method will become a useful complementary method to analyze native chromatin architectures. Further studies of native chromatin structures are awaited.

## 4 Experimental Procedures

### 4.1 Preparation of Mono‐ and Poly‐Nucleosomes From Human HeLa Cells

HeLa S3 cells (ATCC, CCL‐2.2), grown in DMEM supplemented with 10% FBS at 37°C under a 5% CO_2_ atmosphere, were collected from cell culture dishes using a cell scraper. HeLa nuclei were isolated using NE‐PER Nuclear and Cytoplasmic Extraction Reagents (Thermo Fisher Scientific) and crosslinked by 1% formaldehyde at 25°C for 5 min. The crosslinking reaction was quenched by adding 2 M glycine to a final concentration of 150 mM, and the sample was incubated at 25°C for 10 min. Fixed HeLa nuclei were collected by centrifugation at 16,000 × *g* at 4°C for 5 min and resuspended in 500 μL of buffer (10 mM Tris–HCl [pH 8.0], 200 mM KCl, 1 mM CaCl_2_, and 0.5% NP‐40). For the preparation of mono‐nucleosomes, HeLa chromatin from 1.4 × 10^7^ cells was fragmented by sonication using a VP‐050 N ultrasonic homogenizer (TAITEC) at 20% PWM at room temperature for 18 s (3 s × 6 cycles) and then digested with 2510 units of MNase (New England Biolabs). For the preparation of poly‐nucleosomes, HeLa chromatin from 2.0 × 10^7^ cells was fragmented by sonication as described above, but at 25% PWM at room temperature for 9 s (3 s × 3 cycles) and then digested with 50.2 units of MNase (New England Biolabs). MNase digestion was performed at 37°C for 40 min, and quenched by adding 0.5 M EDTA to a final concentration of 10 mM, and then the samples were centrifuged at 15,000 × *g* at 4°C for 5 min. The supernatants were applied onto the top of a 10%–50% sucrose density gradient (11.3 mL in an Ultra‐Clear Tube [Beckman Coulter, 14 × 89 mm], prepared in 10 mM HEPES‐NaOH [pH 7.5], 30 mM NaCl, and 1 mM dithiothreitol) and centrifuged at 35,000 rpm at 4°C for 21 h in an SW 41 Ti rotor (Beckman Coulter). After the centrifugation, 640–660 μL aliquots were collected from the top of the gradient and analyzed by 1.5% agarose gel electrophoresis in 1 × TAE buffer, with or without deproteinization, followed by ethidium bromide staining. For the deproteinization of HeLa chromatin, the fractions (20 μL each) were treated with 9.45 μg of Proteinase K (TAKARA) and 0.08% SDS and incubated at 37°C for 15 min. The fractions containing HeLa nucleosomes with similar DNA lengths were combined, and the buffer was exchanged using a PD‐10 column (Cytiva) to the final sample buffer (10 mM Tris–HCl [pH 7.5], 30 mM NaCl, and 1 mM dithiothreitol). The samples were concentrated with an Amicon Ultra‐4 centrifugal filter unit (Merck, 30,000 MWCO) and used for grid preparation. The protein components of the HeLa poly‐nucleosomes were separated by 16% SDS‐polyacrylamide gel electrophoresis, and the resulting bands were analyzed by mass spectrometry.

### 4.2 Preparation of Uncrosslinked Nucleosome Samples From Human HeLa Cells

The nuclei of HeLa S3 cells (ATCC, CCL‐2.2) were isolated using NE‐PER Nuclear and Cytoplasmic Extraction Reagents (Thermo Fisher Scientific), and collected by centrifugation at 16,000 × *g*, at 4°C for 5 min. HeLa chromatin from 2 × 10^7^ cells was resuspended in 500 μL of 10 mM Tris–HCl (pH 8.0) buffer, containing 200 mM KCl, 1 mM CaCl_2_, and 0.5% NP‐40, and digested with 50.2 units of MNase (New England Biolabs) at 37°C for 40 min. The MNase reaction was quenched by adding 0.5 M EDTA to a final concentration of 10 mM, and then the samples were centrifuged at 15,000 × *g*, at 4°C for 5 min. The subsequent processes (sucrose density gradient ultracentrifugation, fractionation, buffer exchange, and concentration of HeLa chromatin) were performed using the same methods described above.

### 4.3 Grid Preparation and Data Collection for Cryo‐EM Single Particle Analysis

The HeLa nucleosome sample (2.5 μL) was applied to a glow‐discharged grid (Quantifoil R1.2/1.3 200‐mesh Cu). The grid was blotted for 8 s at 4°C under 100% humidity, and then plunged into liquid ethane using a Vitrobot Mark IV (Thermo Fisher Scientific). Cryo‐EM data of the HeLa nucleosome sample were collected using the EPU software (Thermo Fisher Scientific) on a Krios G4 microscope (Thermo Fisher Scientific) equipped with an energy‐filtered K3 detector, operating at 300 kV at a magnification of 81,000× (pixel size of 1.06 Å). For the collection of the HeLa mono‐nucleosome data, all images containing 50 movie frames were recorded with defocus ranging from −1.0 to −2.5 μm and a 10‐s exposure time in the electron counting mode, on an energy‐filtered K3 direct electron detector (Gatan), at a total dose of 58.508 electrons per Å^2^. For the collection of the HeLa nucleosomes in poly‐nucleosomes data, all images containing 40 movie frames were recorded with defocus ranging from −1.0 to −2.5 μm and a 10‐s exposure time in the electron counting mode, using an energy‐filtered K3 direct electron detector (Gatan), at a total dose of 58.094 electrons per Å^2^.

### 4.4 Structure Determination of HeLa Mono‐Nucleosome by Cryo‐EM Single Particle Analysis

In total, 8586 movies were aligned by MOTIONCOR2 (Zheng et al. 2017) with dose weighting, and the contrast transfer function (CTF) was estimated using CTFFIND4 (Rohou and Grigorieff 2015) from digital micrographs. Based on good CTF fit correlation, 6910 micrographs were selected, and all subsequent processing was performed with Relion 3.1 (Zivanov et al. 2018). After Laplacian of Gaussian (LoG)‐based auto‐picking, picked particles were subjected to 2D classification. Three 2D class averages with nucleosome structures were used as references for the following particle picking. After reference‐based auto‐picking, picked particles were subjected to two rounds of 2D classification to discard junk particles. The selected particles were then subjected to five rounds of 3D classification, using the crystal structure of a canonical nucleosome (PDB: 3LZ0) (Vasudevan et al. 2010) as the reference model, with low‐pass filtering of 60 Å. The best 3D class was subjected to 3D auto‐refinement and used for training a Topaz (Bepler et al. 2019) particle‐picking model. Using this model, 3,062,600 particles were picked and subjected to three rounds of 2D classification. After removing junk particles, 1,587,277 particles were subjected to four rounds of 3D classification, using the crystal structure of a human canonical nucleosome (PDB: 3AFA) (Tachiwana et al. 2010) as the reference model, with low‐pass filtering of 60 Å, and 1,050,958 particles were selected and processed by Bayesian polishing and CTF refinement. The resulting particles were subjected to 3D auto‐refinement and post‐processing, and the final cryo‐EM map of the HeLa nucleosome with a resolution of 3.1 Å was obtained. The resolution of the HeLa nucleosome was estimated by the “gold standard” Fourier shell correlation (FSC) at an FSC = 0.143 (Scheres 2016). The figures of the HeLa nucleosome were created by UCSF ChimeraX (Goddard et al. 2018).

### 4.5 Structure Determination of HeLa Mono‐Nucleosome in Poly‐Nucleosomes by Cryo‐EM Single Particle Analysis

In total, 8408 movies were aligned by MOTIONCOR2 (Zheng et al. 2017) with dose weighting, and the CTF was estimated using CTFFIND4 (Rohou and Grigorieff 2015) from digital micrographs. Based on good CTF fit correlation, 7504 micrographs were selected, and all subsequent processing was performed with Relion 3.1 (Zivanov et al. 2018). After LoG‐based auto‐picking in 30 selected micrographs, picked particles were subjected to 2D classification. Three 2D class averages of nucleosome structures were used as references for reference‐based auto‐picking, and 3,778,210 particles were picked from 7504 micrographs and subjected to three rounds of 2D classification to discard junk particles. The 1,111,442 selected particles were subjected to four rounds of 3D classification, using the crystal structure of a canonical nucleosome (PDB: 3AFA) (Tachiwana et al. 2010) as the reference model, with low‐pass filtering of 60 Å. Each of the three selected classes was subjected to 3D auto‐refinement and post‐processing, and the final cryo‐EM maps of the HeLa nucleosome classes 1, 2, and 3 were obtained. The resolutions of the HeLa nucleosome classes 1, 2, and 3 were estimated at 4.09, 3.89, and 3.64 Å, respectively, by the “gold standard” FSC at an FSC = 0.143 (Scheres 2016). The figures of the HeLa nucleosomes were created by UCSF ChimeraX (Goddard et al. 2018). In Figure 2F, HeLa nucleosome maps ad‐hoc low‐pass filtered at 3.6 Å are presented.

### 4.6 Cryo‐ET of Poly‐Nucleosomes

As preparation for plunge‐freezing, HeLa poly‐nucleosomes were mixed with 20‐fold concentrated BSA Gold Tracer (6 nm, AURION) (in final sample buffer: 10 mM Tris–HCl [pH 7.5], 30 mM NaCl, and 1 mM dithiothreitol) at a 5:1 volume ratio. A 2.5 μL portion of the specimen was applied to a glow‐discharged grid (Quantifoil R1.2/1.3 200‐mesh Cu), which was plunge‐frozen in liquid ethane using a Vitrobot Mark IV (Thermo Fisher Scientific). The Vitrobot blotting chamber conditions were set to 4°C and 100% humidity, and the blot time was 6 s.

Cryo‐ET was performed on a Krios G4 microscope (Thermo Fisher Scientific), equipped with a Thermofisher Volta phase plate and a Gatan postcolumn energy filter, and operated at an accelerated voltage of 300 kV. Data were recorded using a direct detector camera (K3, Gatan) operated in the counting mode. Tilt‐series images were collected by a dose‐symmetric tilt scheme (Hagen et al. 2017) using the Tomography 5 software (Thermo Fisher Scientific). In total, 41 tilt‐series images were collected with the following parameters: magnification 53,000×, resulting in a pixel size of 1.7 Å at the specimen level; tilt range of ±60°; tilt increment 3°; start tilt angle 0°; energy filter slit width 20 eV; and total dose ~126 e^−^/Å^2^. For data acquisition, images were collected with a Volta phase plate with −0.5 μm defocus.

Movie frames were aligned and summed with motion correction by the Inspect3D software (Thermo Fisher Scientific). The motion‐corrected tilt‐series images were aligned based on fiducial gold markers and localized using the IMOD package (Kremer et al. 1996). The 8‐times binned tomograms (512 × 720 × 125 pixels volume) were then reconstructed by weighted back projection (WBP).

### 4.7 Template Matching

A human nucleosome structure (PDB: 7VZ4) was downloaded from the Protein Data Bank (PDB), and the Chimera software (35) was used to prepare a density map with a voxel size of 1.7 Å. A template and a mask for template matching of nucleosomes were prepared by pytom‐match‐pick (Chaillet et al. 2023). The template for the nucleosome was prepared from the generated density map of the human nucleosome structure by pytom_create_template.py with the parameters: output voxel size 13.6; center; CTF correction; defocus 0.5; amplitude contrast 0.07; voltage 300; Cs 2.7; flip phase invert; box size 64. The spherical mask for template matching was prepared by pytom_create_mask.py with the parameters: box size 64; voxel size 13.6; radius 6; sigma 1. The template and mask were used for template matching with eight times binned tomograms, using the parameter of angular search 7 in pytom‐match‐pick (Chaillet et al. 2023). After the template matching, 12,693 particles were extracted from five tomograms. The coordinates of the extracted particles for eight times binned tomograms were calibrated for the unbinned tomograms.

### 4.8 Data Processing and Subtomogram Averaging of Poly‐Nucleosomes

Initial defocus and phase shift estimation per tilt image for subtomogram averaging were performed by CTFFIND4 (Rohou and Grigorieff 2015). The particle coordinates of the nucleosome picked by PyTom template matching were imported to RELION‐4.0.1 (Zivanov et al. 2022). The alignment of particles was carried out by RELION‐4.0.1 (Zivanov et al. 2022). The detailed workflow of subtomogram averaging is shown in Figure S4. Following the initial 3D classification, 8545 particles were selected and subjected to refinement for mapping back (Figures 4 and S4, right column). For fitting the nucleosome atomic model, 1334 particles were selected to obtain a 3D volume through several rounds of 3D classification (Figures 3B,C and S4, left column). The pixel size of the nucleosome volumes was adjusted to 1.63 Å/pix to match the scale of the nucleosome atomic model.

### 4.9 Mapping Back of Nucleosomes in the Tomograms

Mapping back of nucleosomes was performed using ArtiaX in ChimeraX (Ermel et al. 2022). The clustering of nucleosome coordinates in the tomograms was conducted based on the DBSCAN algorithm (Ester et al. 1996). Particles located within a radius of 15 nm were grouped into the same class, and classes containing three or more particles were visualized in Figures 4 and S5.

### 4.10 Protein Identification by LC–MS/MS Analysis

HeLa chromatin fragments were separated by SDS‐PAGE and visualized with Oriole fluorescent gel stain (Bio‐Rad). Target proteins in the excised gel slices were in‐gel digested with trypsin and identified by nano LC–MS/MS analysis, following a modified procedure from previous studies (Shevchenko et al. 1996; Funato et al. 2018; Hatazawa et al. 2022). The LC–MS/MS analysis was conducted using an LTQ‐Orbitrap Fusion mass spectrometer equipped with an Ultimate3000 nano LC containing an autosampler (Thermo Fisher Scientific). The nano LC gradient was delivered at 300 nL/min and consisted of a linear gradient from 2% to 40% acetonitrile over 60 min. The 10 most intense precursor ions were selected during MS1 scans and subsequently fragmented by higher‐energy collisional dissociation in MS2 scans. These data were searched against the *Homo sapiens* protein sequence database from UniProt through the search program application Proteome Discoverer 2.1 (Thermo Fisher Scientific) for protein identification. Spectra were searched with a precursor mass tolerance of 10 ppm and a fragment mass tolerance of 0.6 Da, allowing up to two missed cleavage sites. Carbamidomethylation of cysteine was set as a fixed modification, while oxidation of methionine and phosphorylation of serine, threonine, and tyrosine were set as variable modifications.

### 4.11 The Use of Large Language Models

ChatGPT was used for grammatical corrections in the text and for providing the Python code to perform DBSCAN.

## Author Contributions

**Suguru Hatazawa:** methodology, formal analysis, investigation, resources, writing – review and editing, visualization, funding acquisition. **Yoshiyuki Fukuda:** methodology, formal analysis, writing – review and editing, visualization. **Yuki Kobayashi:** formal analysis, investigation, resources. **Lumi Negishi:** methodology, formal analysis, investigation. **Masahide Kikkawa:** writing – review and editing, funding acquisition, project administration. **Yoshimasa Takizawa:** formal analysis, writing – review and editing, data curation, funding acquisition. **Hitoshi Kurumizaka:** conceptualization, visualization, supervision, funding acquisition, writing – original draft, writing – review and editing, project administration.

## Conflicts of Interest

The authors declare no conflicts of interest.

## Supporting information


**Data S1.** Supporting Information.

## Data Availability

The cryo‐EM reconstructions of HeLa nucleosomes have been deposited in the Electron Microscopy Data Bank (EMDB) under the following accession codes: EMD‐63078, EMD‐63079, EMD‐63080, and EMD‐63081. The subtomogram‐averaged maps of HeLa nucleosomes in poly‐nucleosomes have been deposited in the EMDB under the following accession codes: EMD‐63144 and EMD‐63145.
