# Supplementary material for: Cryo‐EM Structures of Native Chromatin Units From Human Cells
Source: Genes Cells. 2025 Apr 14;30(3):e70019. doi: 10.1111/gtc.70019 (PMC11995444; doi:10.1111/gtc.70019)
Supplement: Supplementary file 1 — Data S1. Supporting Information. [file GTC-30-0-s001.pdf]

Supporting information

Cryo-EM structures of native chromatin units from human cells

Suguru Hatazawa[1], Yoshiyuki Fukuda[2], Yuki Kobayashi[1], Lumi Negishi[1],

Masahide Kikkawa[3], Yoshimasa Takizawa[1,4,*], and Hitoshi Kurumizaka[1,5,6,*]

[1]Laboratory of Chromatin Structure and Function, Institute for Quantitative Biosciences, The University of Tokyo, 1-1-1 Yayoi, Bunkyo-ku, Tokyo 113-0032, Japan.

[2]Division of Molecular CytoMorphology, Institute of Advanced Medical Sciences, Tokushima University, 3-18-15 Kuramoto-cho, Tokushima, Tokushima 770-8503, Japan.

[3]Department of Cell Biology and Anatomy, Graduate School of Medicine, The University of Tokyo, 7-3-1 Hongo, Bunkyo-ku, Tokyo 113-0033, Japan.

[4]Department of Computational Biology and Medical Sciences, Graduate School of Frontier Sciences, The University of Tokyo, 1-1-1 Yayoi, Bunkyo-ku, Tokyo 113-0032, Japan.

[5]Department of Biological Sciences, Graduate School of Science, The University of Tokyo, 1-1-1 Yayoi, Bunkyo-ku, Tokyo 113-0032, Japan.

[6]RIKEN Center for Biosystems Dynamics Research, 1-7-22 Suehiro-cho, Tsurumi-ku, Yokohama 230-0045, Japan.

*Correspondence should be addressed to ytakizawa@iqb.u-tokyo.ac.jp (Y.T.), kurumizaka@iqb.u-tokyo.ac.jp (H.K.)

Figure S1. Gel electrophoretic analyses of native chromatin fragments from the HeLa cell nuclear extract

Figure S2. Cryo-EM data processing of the HeLa mono-nucleosome

Figure S3. Cryo-EM data processing of the HeLa nucleosome in poly-nucleosomes

Figure S4. Workflow for subtomogram averaging of the HeLa nucleosome

Figure S5. 3D organization of nucleosomes in tomograms

Figure S6. Representative cryo-EM micrograph of uncrosslinked HeLa poly-nucleosomes

Table S1. Cryo-EM data collection and image processing for HeLa nucleosomes

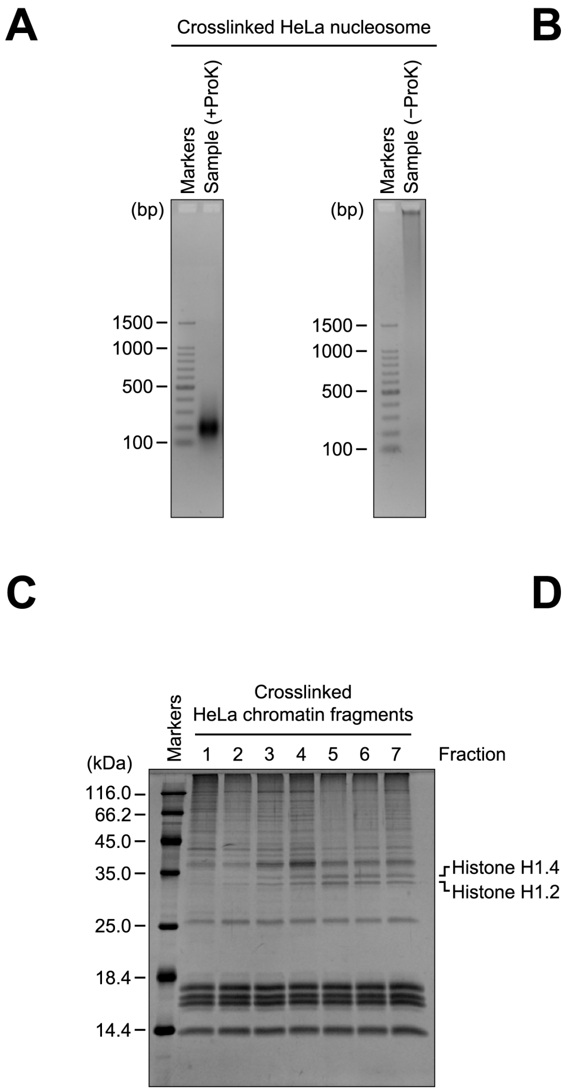

Figure S1. Polyacrylamide and agarose gel electrophoretic analyses of native chromatin fragments from the HeLa cell nuclear extract. (**A**) Agarose gel electrophoretic analyses of crosslinked HeLa mono-nucleosomes fractionated by sucrose gradient ultracentrifugation. The sample was analyzed by agarose gel electrophoresis with (left) or without (right) deproteinization. DNA was stained with ethidium bromide. (B) SDS-polyacrylamide gel electrophoresis of the HeLa mono-nucleosomes. The nucleosomes were analyzed by 16% SDS-polyacrylamide gel

electrophoresis with Oriole fluorescent gel staining. (C) SDS-polyacrylamide gel electrophoresis of chromatin fragments from HeLa cells. The chromatin fragments fractionated by sucrose gradient ultracentrifugation were analyzed by 16% SDS-polyacrylamide gel electrophoresis with Oriole fluorescent gel staining. Linker histones H1.2 and H1.4 were identified by mass spectrometry. (D) Agarose gel electrophoretic analyses of uncrosslinked HeLa nucleosomes fractionated by sucrose gradient ultracentrifugation. The sample was analyzed by agarose gel electrophoresis with (left) or without (right) deproteinization. DNA was stained with ethidium bromide.

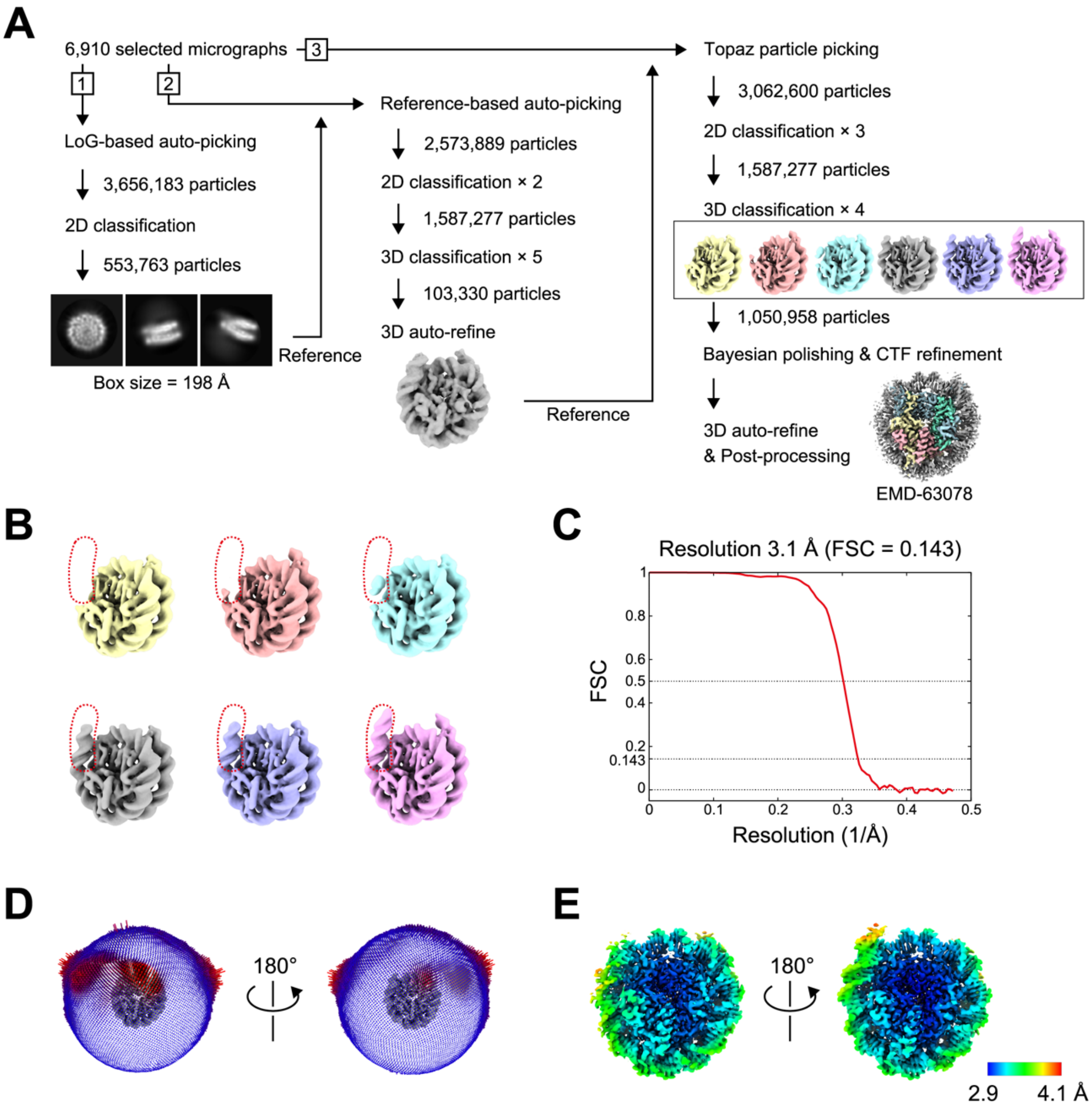

Figure S2. Cryo-EM data processing of the HeLa mono-nucleosome. (**A**) Workflow for cryo-EM data processing of the HeLa mono-nucleosome. (**B**) 3D classes of HeLa mono-nucleosomes derived from single-particle analysis. The entry-exit region of the nucleosomal DNA, which exhibits a distinct structure in each 3D class, is surrounded by red dotted lines. (**C**) Gold-standard Fourier Shell Correlation (FSC) curve of the HeLa mono-nucleosome. (**D**) Euler angular distribution of the HeLa mono-nucleosome particles used in the final 3D reconstruction. (**E**) Local

resolution assessment of the HeLa mono-nucleosome, showing the resolution range across the map from 2.9 Å to 4.1 Å.

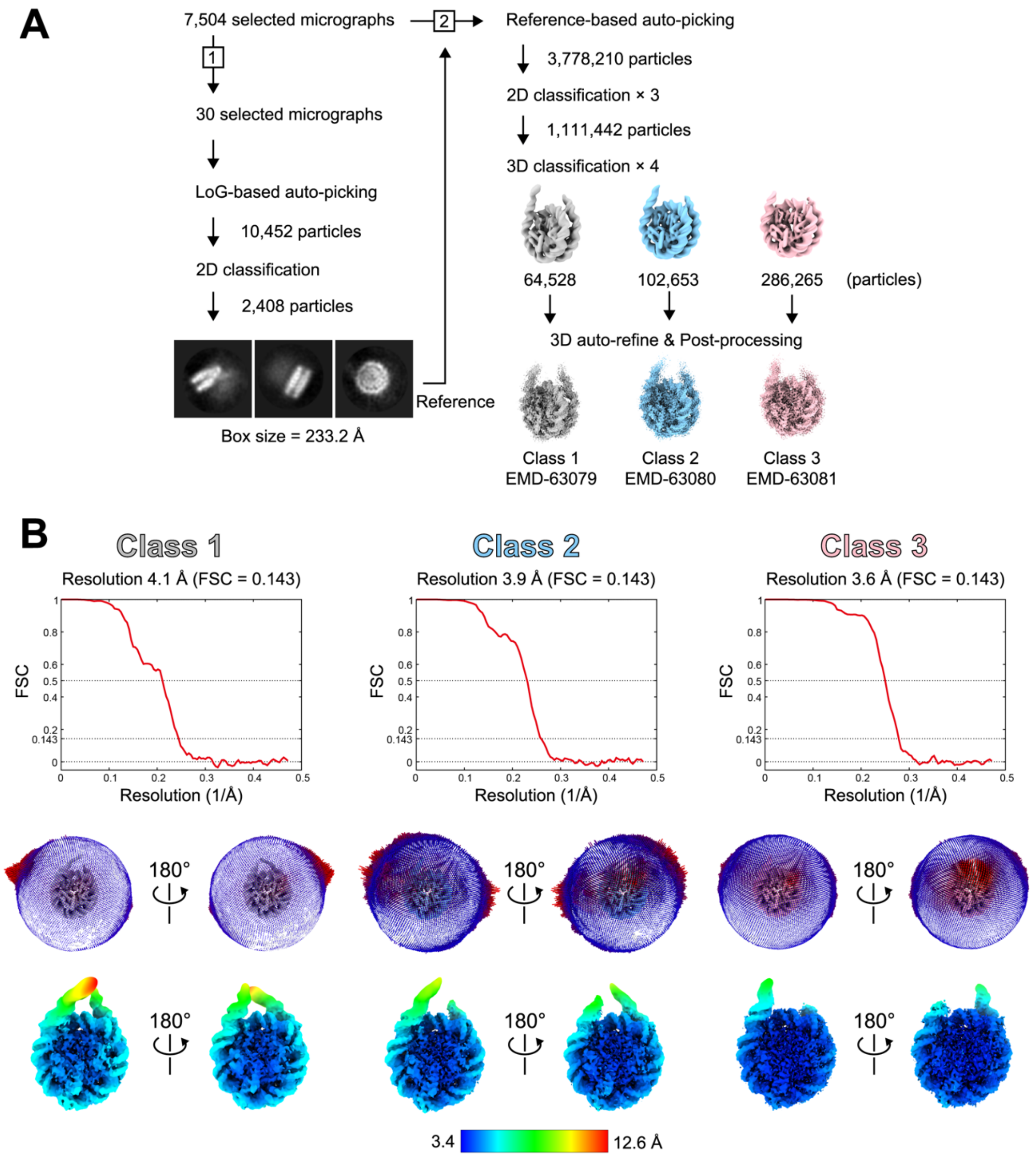

Figure S3. Cryo-EM data processing of the HeLa nucleosome in poly-nucleosomes. (**A**) Workflow for cryo-EM data processing of the HeLa nucleosome in poly-nucleosomes. (**B**) Gold-standard FSC curves, local resolution assessments of the HeLa nucleosome in poly-nucleosomes,

and Euler angular distributions of the HeLa nucleosome particles used in the final 3D reconstruction. The local resolution across the HeLa nucleosomes ranges from 3.4 Å to 12.6 Å.

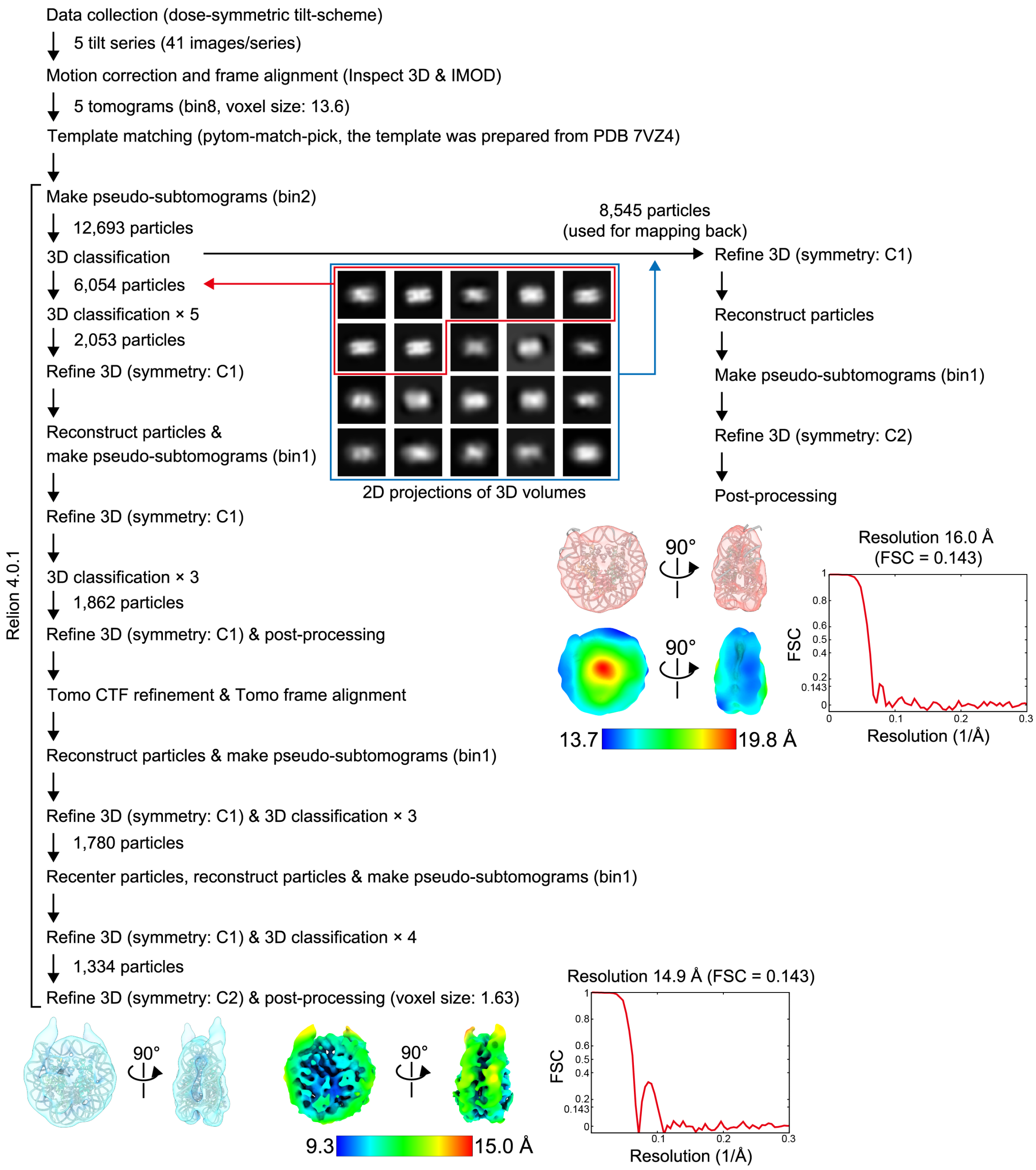

Figure S4. Workflow for subtomogram averaging of the HeLa nucleosome. Superimposed views (the atomic model of the reconstituted nucleosome (PDB ID: 7VZ4) into the subtomogram averages), the gold-standard FSC curves, and local resolution assessments of the subtomogram averages are displayed.

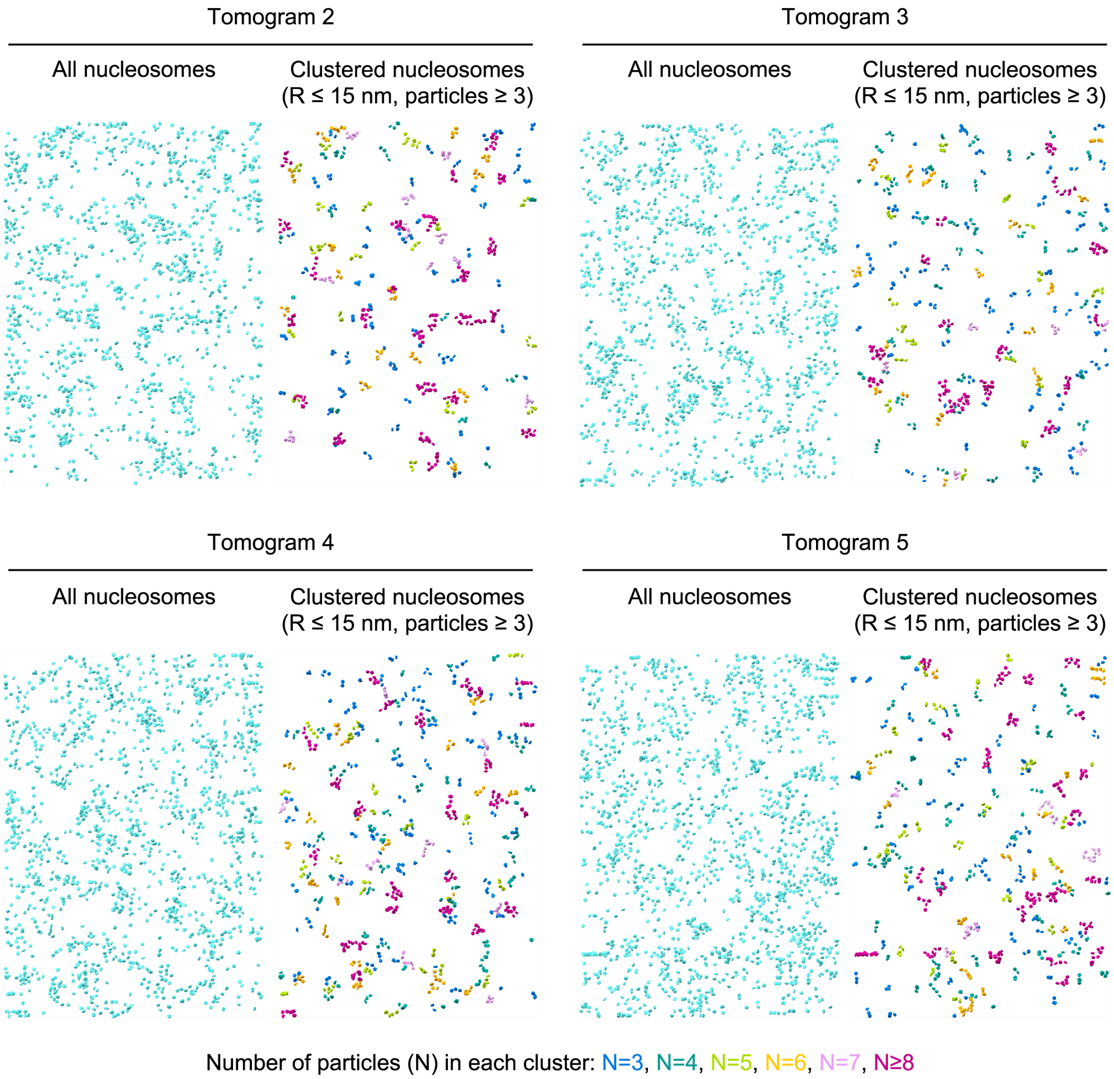

Figure S5. 3D organization of nucleosomes in tomograms. Mapping back of all nucleosomes and the clustered nucleosomes is displayed in the tomograms. Each class of nucleosomes is colored based on the number of particles in the cluster.

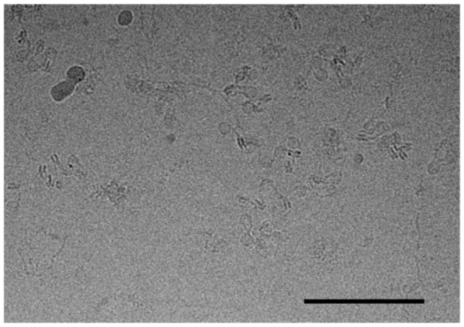

Figure S6. Representative cryo-EM micrograph of uncrosslinked HeLa poly-nucleosomes. Scale bar indicates 100 nm.

Table S1. Cryo-EM data collection and image processing for HeLa nucleosomes

	HeLa mono-nucleosome	HeLa nucleosome in poly-nucleosomes		
		Class 1	Class 2	Class 3
Accession code	EMD-63078	EMD-63079	EMD-63080	EMD-63081
**Data collection**				
Electron microscope	Krios G4			
Camera	K3			
Magnification	81,000×			
Voltage (kV)	300			
Electron exposure (e–/Å^2)	58.508	58.094		
Exposure time (second)	10			
Defocus range (μm)	1.0 to 2.5			
Pixel size (Å)	1.06			
Movie frames (no.)	50	40		
Total micrographs (no.)	8,586	8,408		
**Reconstruction**				
Software	Relion 3.1			
Initial particle images (no.)	3,062,600	3,778,210		
Final particle images (no.)	1,050,958	64,528	102,653	286,265
Symmetry imposed	C1			
Map resolution (Å)	3.1	4.09	3.89	3.64
Map resolution range (Å)	2.90 to 10.04	3.90 to 14.11	3.69 to 13.15	3.47 to 10.52
Map sharpening B factor (Å^2)	-137.492	-141.627	-157.056	-164.357
